# PARQVE: PROJECT ARTHRITIS RECOVERING QUALITY OF LIFE THROUGH EDUCATION: TWO-YEAR RESULTS

**DOI:** 10.1590/1413-785220172501165604

**Published:** 2017

**Authors:** MÁRCIA UCHOA DE REZENDE, RENATO FRUCCHI, ALEXANDRE FELÍCIO PAILO, GUSTAVO CONSTANTINO DE CAMPOS, THIAGO PASQUALIN, MARCELO ISSAO HISSADOMI

**Affiliations:** 1. Universidade de São Paulo, Faculdade de Medicina, Hospital das Clínicas, Instituto de Ortopedia e Traumatologia, São Paulo, SP, Brazil.

**Keywords:** Osteoarthritis, Knee, Quality of life, Patient education as topic, Treatment outcome

## Abstract

**Objective:**

To evaluate the effects of a multi-professional educational program in patients with knee osteoarthritis (KOA)***.***

**Methods:**

Prospective randomized controlled trial with 195 KOA patients. One group was submitted to two-day lectures and received educational material about KOA (Class group), and the control group received the educational material only. Patients were evaluated at baseline, twelve and 24 months. At evaluation, patients answered pain and functional questionnaires (WOMAC, Lequesne, VAS and SF-36); reported the intensity of exercise per week; measured the body fat percentage, weight and height to estimate body mass index (BMI); and performed Timed Up & Go (TUG) and Five-Times-Sit-to-Stand (FTSST) tests***.***

**Results:**

The groups presented similar results in all time points with respect to pain (VAS and WOMAC pain), WOMAC, BMI and body fat percentage (p>0.05). The Class group exhibited improved function according to the Lequesne questionnaire, whereas the control group worsened (p=0.02) during follow-up (p<0.02). TUG (p=0.01) and FTSST (p<0.001) improved in the Class group. A higher percentage of patients in the Class group performed regular physical activity (p=0.045)***.***

**Conclusions:**

The educational program with classes improved the consistency of physical activity and the subjective and objective function of patients with KOA.***Level of evidence IA, Prospective Randomized Controlled Trial.***

## RESUMO

**Objetivo:**

***Avaliar o efeito de um programa educacional multiprofissional para pacientes com gonartrite.***

**Métodos:**

***Trata-se de um estudo prospectivo randomizado com 195 pacientes portadores de gonartrite. Um grupo recebeu material didático sobre osteoartrite (grupo Controle) e o outro grupo participou de dois dias de aulas práticas e teóricas sobre osteoartrite e também recebeu o material didático (grupo Aula). Os pacientes preencheram questionários para avaliar dor, função e qualidade de vida (WOMAC, Lequesne, EVA, SF-36) no momento da inclusão, e após 12 e 24 meses de seguimento. Nestes mesmos períodos, os pacientes tiveram calculados a percentagem de gordura e o índice de massa corpórea (IMC); realizaram os testes de senta e levanta (TSL) e "Timed-Up-and-Go" (TUG), além de responder perguntas sobre a intensidade da atividade física semanal realizada.***

**Resultados:**

***Os dois grupos não diferiram quanto aos resultados de dor, função (WOMAC), IMC e porcentagem de gordura corpórea (p>0,05). O grupo Aula melhorou e o Controle piorou a função pelo Lequesne (p=0,02) ao longo do tempo (p<0,02). O TUG (p=0,01) e o TSL (p<0,001) melhoraram principalmente no grupo Aula. Uma porcentagem maior de pacientes do grupo Aula aderiu à atividade física regular (p=0,045) .***

**Conclusão:**

*O programa educacional com aulas melhora adesão à atividade física e a função subjetiva e objetiva dos pacientes com gonartrite.* ***Nível de Evidência IA, Estudo Prospectivo Controlado e Randomizado.***

## INTRODUCTION

The incidence of osteoarthritis (OA) is known to increase with longevity, obesity and low socioeconomic level.^1^ ^,^ ^2^

Obesity and longevity are increasing in Brazil.^3^ ^,^ ^4^ Approximately 50.2% of Brazilians have no education or incomplete primary education,^5^ and although the GDP per capita in Brazil was R$ 20,876,00 reais (roughly US $ 8,134.00 dollars) in 2015,^6^ people who earned more than 10 times the minimum monthly wage (minimum wage being approximately US$ 300.00) represented only 3.1% of the employed population in the country in 2010.^5^ Therefore, the number of patients with OA is expected to increase in Brazil. Thus, a program that aims to change the fate of OA patients by decreasing Body Mass Index (BMI), increasing physical activity and providing tools to enhance their quality of life is essential to alleviate such a burden to society.^7^

The optimal management of OA requires a combination of pharmacological and non-pharmacological modalities.^8^ There are several reports of minor effects of educational programs on pain, function, time spent in gyms and weight loss.^8^ A previous positive experience in a weekly educational program on patients with osteoporosis inspired the present proposal of two days of lectures and workshops about OA to patients with knee OA (KOA) that are reinforced by telephone calls. Our one-year results failed to show a relevant difference in the groups that received telephone calls.^8^ Because these telephone calls were time-consuming, they were suspended in the second year. This study evaluated the two-year effects of a multi-professional conservative treatment for patients with KOA by comparing the offering of the educational program with or without classes by assessing the subjective pain, function and quality of life questionnaires and by objective measures of (BMI), percentage of body fat (PBF), functional tests and engagement in regular physical activity.

## METHODS

This study was performed at the Department of Orthopedics and Traumatology, São Paulo, Brazil, after receiving approval from the Ethics Committee for the Analysis of Research Projects (CAPPesq) under protocol number 0622/11. 

Clinical trials registration number: NCT01572051.

This was a prospective, randomized controlled trial. This study followed the guidelines of the CONSORT statements for randomized controlled trials and non-drug treatments.

The care providers included six orthopaedic surgeons, four psychologists, three social workers, one nutritionist, five occupational therapists, three physical therapists and two physical educators, all of whom were volunteers or staff at the Orthopaedic Institute, Hospital das Clínicas, University of São Paulo.

Patients had to meet the following criteria: outpatient aged 45 years or older with KOA according to the American College of Rheumatology clinical and radiological definition;^9^ no rheumatoid arthritis or any rheumatologic disease other than OA; had received typical care for OA in the past six months; knee pain rated above 30 mm on a numerical scale and necessitating drug treatment without any neurological problems; and able to understand and provide informed consent. The exclusion criteria included undergoing surgery during the study that was not related to OA and would prevent daily regular exercises, participating in another program with nutritional education or engaging in another clinical trial. Patients who were not able to perform the functional tests at baseline were excluded only from the functional analysis.

Participants were patients undergoing typical care for the treatment of KOA at the Osteometabolic Diseases Group, Department of Orthopedics and Traumatology, Hospital das Clínicas, University of São Paulo. By January 2012, 306 patients were undergoing typical care for KOA as described.^7^

At enrolment, patients were asked to respond to the VAS (Visual Analogue Scale), WOMAC(tm), Lequesne, and SF-36 questionnaires and to assess the frequency and intensity of physical activity performed/week.^10^ ^-^ ^12^ Weight, height, and seven skin folds were measured to calculate body mass index (BMI) and percentage of body fat (PBF). Patients were asked to perform timed up-and-go TUG and five times sit to stand FTSST tests.^13^ ^,^ ^14^ All patients received a plain radiograph of their knees, including weight-bearing anterior-posterior, lateral and patellar axial views. Three orthopaedic surgeons examined all radiographs to classify the severity of OA according to Kellgren and Lawrence (K&L)^15^ In case of disagreement between two surgeons, the third surgeon was decisive. 

Participants were randomly allocated into eight subgroups (1 to 4, according to the intervals between days of lectures, and A and B, according to the use or absence of telephone calls) of 28 or 29 participants each. The Class group had six subgroups, named 1, 2, and 3, which had lectures one, two and three months apart, respectively, either with (A) or without (B) bimonthly telephone calls. Subgroup 4 (with (A) and without (B) bimonthly telephone calls) received the educational material only and formed the Control group. Patients in each Class subgroup were asked to come to the hospital on two specific Saturdays according to the intervals of each group to participate in the educational program.^7^

All participants received the written and video (DVD) information of the lectures given on the first day of class.^7^ The DVD was 2 hours and 23 minutes long. Patients from subgroups 4A and 4B watched the DVD for the first time at the hospital. All patients were asked to read the text and/or watch the DVD at home at least three times.

The physicians called patients in subgroup A two months after the lecture and every other month until the 1-year reassessment to reinforce the information given in the educational program. 

Twelve and 24 months after the final lecture or after receiving the educational material, the patients returned for evaluation, where the same assessments performed at baseline were repeated.

### Sample

This was a pilot study to evaluate the effectiveness of a two-day program about OA with respect to the sole offering of educational material (booklet and DVD) with particular intervals between classes and telephone calls. The authors aimed for 30 patients in each subgroup.

Randomization was performed by a computer-generated program available at http://www.randmization.com/. 

### Blinding

There was no difference in the demographic information between the groups. Groups 1 to 3 received classroom instruction from all professionals and both audio-visual and written instructions, which group 4 also received. When signing the informed consent forms, the patients knew that the groups would differ according to the time between classes, lack of classes and telephone calls. Evaluators did not know to which group the patient belonged. Two main assistants scheduled appointments and classes, retrieved material, and plotted the questionnaires' results in Excel sheets. 

### Statistical analysis

The nominal characteristics were described for each group using the absolute and relative frequencies, and the existence of associations between groups and features was verified using the chi-square test and the likelihood ratio for race. OA severity was compared between groups using the Kruskal-Wallis test. Quantitative characteristics were described for the groups using summary measures and were compared between groups using Analysis of Variance (ANOVA) for repeated measures followed by Tukey's multiple comparison test.

Scores were described according to groups, subgroups and moments of evaluation using summary measures (mean, standard deviation and 95% confidence interval). Values were compared between groups, telephone calls and moments (of assessment) using a three-factor analysis of variance with repeated measures, followed by Tukey's multiple comparison test to compare groups, telephone calls and moments of assessment when needed.

Variations (changes) in the function, pain and quality of life scores were calculated. Changes in the BMI and fat percentage between the two-year follow-up and baseline were also measured. Subsequently, Pearson's correlations were determined between these variations in the scores and between the changes in the scores and the baseline measures to check for relationships to patient improvement. The variations in scores were described using the qualitative characteristics of the patients and compared between categories using Student's t-test or ANOVA.

The tests were performed with a significance level of 5%. All analyses were carried out using SPSS 17.

## RESULTS

**Figure 1:**
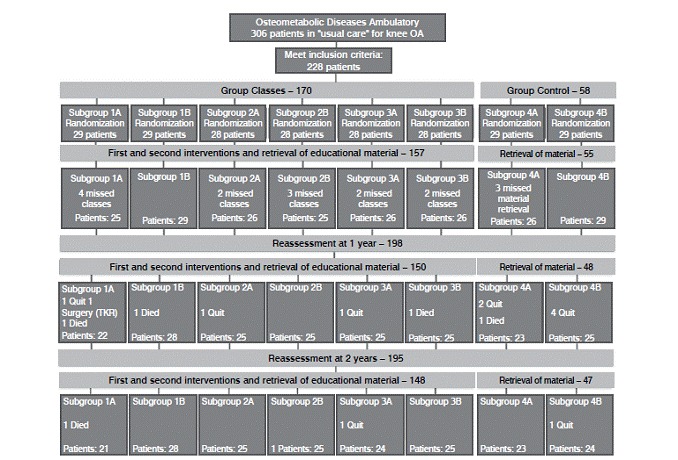
Flow of participants through the trial.

Three hundred six patients were assessed for eligibility, and 246 patients met the inclusion criteria; however, only 228 agreed to participate

(Figure 1). Twenty-eight patients were assigned to each of the subgroups 2A, 2B, 3A and 3B; 29 patients were assigned to each of the remaining groups. Sixteen patients missed classes (because they lost interest, weather conditions prevented access to the hospital or they could not attend classes when scheduled) and were excluded. At this point, the number of patients in each subgroup varied from 25 (1A, 2B) to 29 (1B, 4B). At the one-year reassessment, four patients had died (one each from subgroups 1A, 1B, 3B and 4A), and 1 patient had undergone total knee replacement (1A). Nine patients quit (lost interest): one from subgroup 1A, one from 2A, one from 3A, two from 4A and four from subgroup 4B (Figure 1). At the two-year reassessment, one patient had died (subgroup 1A), one lost interest and quit (subgroup 3A) and one was not able to attend the evaluation because of family problems (4B). In total, 33 patients were lost from the study, of whom 11 were from subgroup 4, 9 from subgroup 1, 7 from subgroup 3, and 6 from subgroup 2.

Subgroups were homogeneous for nominal valued features, such as degree of KOA, age, gender, race, percentage of body fat, years of schooling, affected side or bilaterality, and questionnaire (subjective) results (p>0.05, Tables 1 to 4). The results of WOMAC and the physical and mental components of the SF-36 questionnaires changed timewise but not between subgroups (p=0.007, p=0.020 and p=0.027, respectively). BMI was statistically different between subgroups 2 and 3 (p=0.047), but PBF was not (p=0.464). The latter varied during the study (p<0.001).

**Table 1:** Descriptions of personal and clinical characteristics of patients according to the subgroups and results of statistical tests.

	Subgroup					
Variable	1		2		3		4		p
	n	%	n	%	n	%	n	%	
K&L Right									0.149*
0	0	0.0	0	0.0	2	4.1	0	0.0	
1	4	8.2	1	2.0	6	12.2	1	2.3	
2	20	40.8	17	34.0	12	24.5	13	29.5	
3	15	30.6	21	42.0	19	38.8	14	31.8	
4	10	20.4	11	22.0	10	20.4	16	36.4	
K&L Left									0.361*
0	0	0.0	0	0.0	0	0.0	3	6.4	
1	5	10.2	3	6.1	5	10.2	1	2.1	
2	21	42.9	14	28.6	14	28.6	15	31.9	
3	16	32.7	25	51.0	16	32.7	20	42.6	
4	7	14.3	7	14.3	14	28.6	8	17.0	
Gender									0.893
Male	13	26.5	10	20.0	12	24.5	11	23.4	
Female	36	73.5	40	80.0	37	75.5	36	76.6	
Race									0.616#
White	27	56.2	31	63.3	32	66.7	33	70.2	
Mulatto/Mestizo	12	25.0	11	22.4	10	20.8	8	17.0	
Black	9	18.8	5	10.2	5	10.4	4	8.5	
Asian	0	0.0	2	4.1	1	2.1	2	4.3	
Knee									0.745
Right	39	79.6	43	86.0	41	83.7	41	87.2	
Left	10	20.4	7	14.0	8	16.3	6	12.8	
Bilateral									0.866
No	15	30.6	14	28.0	17	34.7	13	27.7	
Yes	34	69.4	36	72.0	32	65.3	34	72.3	

Chi-sqare test; # Likelyhood ratio test; * Kruskal-Wallis's test; K&L: Kellgren and Lawrence

**Table 2:** Descriptions of quantitative characteristics according to subgroups and the results of statistical tests.

Variable	Subgroup	Mean	SD	95% CI		n	p
				Lower	Upper		
Age (years)	Subgroup 1	64.4	9.7	61.7	67.1	49	0.108
	Subgroup 2	62.1	8.3	59.8	64.4	50	
	Subgroup 3	66.6	9.8	63.9	69.4	49	
	Subgroup 4	64.3	8.7	61.8	66.8	47	
Time of study (years)	Subgroup 1	7.4	2.7	6.6	8.1	49	0.556
	Subgroup 2	7.8	3.3	6.9	8.7	49	
	Subgroup 3	8.0	3.5	7.0	8.9	49	
	Subgroup 4	8.2	2.2	7.6	8.8	47	
BMI (kg/m^2)^	Subgroup 1	31.3	5.2	29.9	32.8	49	0.049
	Subgroup 2	32.8	6.0	31.2	34.5	50	
	Subgroup 3	29.8	4.7	28.5	31.1	49	
	Subgroup 4	31.3	5.2	29.8	32.8	46	
BFP	Subgroup 1	35.8	9.4	33.2	38.4	49	0.421
	Subgroup 2	37.8	7.6	35.7	40.0	50	
	Subgroup 3	35.1	8.2	32.8	37.4	49	
	Subgroup 4	36.3	8.4	33.8	38.7	47	
ANOVA							

SD: Standard Deviation / CI: Confidence Interval / BMI: Body Mass Index / BFP: Body Fat Percentage.

**Table 3:** Descriptions of functional (WOMAC and Lequesne) and pain (WOMAC pain) scales according to subgroups and moments.

Subgroup	Calling in the first year		WOMAC			WOMAC Pain			Lequesne		
			Baseline	1 year	2 years	Baseline	1 year	2 years	Baseline	1 year	2 years
1	Yes (A)	Mean (SD)	44 (20.2)	39.1 (15)	35.8 (15.9)	8.8 (4.3)	7.5 (3.3)	7.6 (2.9)	11.2 (4.1)	10.6 (3.4)	10.1 (3.8)
		95% CI	(35.3 - 52.6)	(32.7 - 45.5)	(28.8 - 42.8)	(6.9 - 10.6)	(6.1 - 8.9)	(6.3 - 8.9)	(9.5 - 13)	(9.1 - 12.1)	(8.4 - 11.7)
	No (B)	Mean (SD)	48.8 (15.8)	44.3 (14)	45.3 (16.8)	9.1 (4.3)	8.3 (3.6)	9.4 (3.8)	11.9 (4)	12.4 (3.1)	11.7 (4.7)
		95% CI	(43 - 54.7)	(39.1 - 49.5)	(39.1 - 51.5)	(7.5 - 10.7)	(7 - 9.7)	(8 - 10.8)	(10.4 - 13.4)	(11.3 - 13.6)	(10 - 13.4)
2	Yes (A)	Mean (SD)	49 (17.2)	42 (19.5)	43.2 (21)	9.6 (3.2)	7.7 (3.8)	8.5 (4.6)	12.3 (3.4)	11.6 (4.8)	12.4 (4.7)
		95% CI	(42.2 - 55.7)	(34.4 - 49.6)	(34.8 - 51.6)	(8.4 - 10.9)	(6.2 - 9.2)	(6.7 - 10.4)	(10.9 - 13.6)	(9.7 - 13.5)	(10.5 - 14.3)
	No (B)	Mean (SD)	47.2 (19.3)	44.8 (20.4)	39.4 (16.7)	9.9 (4.4)	8.5 (4.2)	7.8 (3.7)	12.5 (4.3)	11.8 (4.7)	11.4 (3.8)
		95% CI	(39.6 - 54.8)	(36.8 - 52.8)	(32.4 - 46.4)	(8.2 - 11.6)	(6.9 - 10.2)	(6.2 - 9.3)	(10.8 - 14.2)	(9.9 - 13.6)	(9.8 - 13)
3	Yes (A)	Mean (SD)	42.8 (19.5)	43.6 (20)	47.3 (21.2)	8.9 (4)	8.5 (3.9)	9.9 (4.3)	11.6 (4.6)	11.8 (4.5)	12.4 (4.4)
		95% CI	(35 - 50.6)	(35.6 - 51.6)	(38.6 - 56)	(7.3 - 10.5)	(7 - 10.1)	(8.2 - 11.6)	(9.7 - 13.4)	(10 - 13.6)	(10.6 - 14.1)
	No (B)	Mean (SD)	43.8 (19)	42.6 (14.5)	39.4 (16.8)	8.3 (4.3)	8.7 (3.2)	7.3 (3.5)	11.2 (3.8)	12.1 (3.5)	10.9 (4.8)
		95% CI	(36.4 - 51.3)	(36.9 - 48.3)	(32.5 - 46.3)	(6.6 - 10)	(7.5 - 10)	(5.8 - 8.7)	(9.7 - 12.7)	(10.7 - 13.4)	(8.9 - 12.8)
4	Yes (A)	Mean (SD)	44.4 (13.8)	47.5 (19)	45.8 (14.9)	9.4 (4.1)	9.6 (4.7)	9.6 (3.4)	11.9 (4.6)	12.2 (4)	12.6 (4.2)
		95% CI	(38.8 - 50.1)	(39.7 - 55.3)	(39.6 - 52)	(7.7 - 11.1)	(7.7 - 11.5)	(8.1 - 11)	(10 - 13.8)	(10.5 - 13.9)	(10.9 - 14.3)
	No (B)	Mean (SD)	42.6 (21.8)	44.9 (20.1)	42 (15.3)	8 (4.2)	9.1 (3.7)	8.6 (3.2)	12.5 (4.4)	12.3 (4.2)	11.7 (4.2)
95% CI	(33.9 - 51.3)	(36.8 - 52.9)	(35.4 - 48.5)	(6.4 - 9.7)	(7.6 - 10.6)	(7.2 - 10)	(10.7 - 14.2)	(10.7 - 14)	(9.9 - 13.5)

SD: Standard Deviation / CI: Confidence Interval.

**Table 4:** Descriptions of pain (VAS) and quality of life (SF-36) scales according to subgroups and moments.

Subgroup	Calling in the first		VAS			SF-36 PCS			SF-36 MCS		
			Baseline	1 year	2 years	Baseline	1 year	2 years	Baseline	1 year	2 years
1	Yes (A)	Mean (SD)	52.3 (25.3)	49.4 (22.2)	48.9 (15.2)	32.3 (8.2)	32.2 (9.1)	36.9 (8.6)	44.4 (12.3)	48.3 (13.1)	49.7 (12.9)
		95% CI	(41.5 - 63.1)	(39.9 - 58.9)	(42.2 - 55.6)	(28.8 - 35.9)	(28.3 - 36)	(33.1 - 40.7)	(39.2 - 49.7)	(42.7 - 53.9)	(44.1 - 55.3)
	No (B)	Mean (SD)	60.6 (24.8)	54.4 (23.2)	53.4 (26.2)	32.6 (8.1)	34.2 (7.3)	34.7 (7.2)	46.6 (14.1)	49.5 (10)	49.2 (13.4)
		95% CI	(51.5 - 69.8)	(45.8 - 63)	(43.6 - 63.1)	(29.6 - 35.6)	(31.5 - 36.9)	(32 - 37.4)	(41.4 - 51.8)	(45.8 - 53.2)	(44.2 - 54.2)
2	Yes (A)	Mean (SD)	67.8 (24.1)	53.4 (23.9)	58.2 (23)	30.3 (6.5)	32 (8.4)	32.5 (6.1)	44 (12.6)	45.8 (14.2)	44.4 (13.6)
		95% CI	(58.3 - 77.2)	(44 - 62.8)	(49 - 67.4)	(27.8 - 32.9)	(28.8 - 35.3)	(30.1 - 35)	(39.1 - 49)	(40.2 - 51.4)	(39 - 49.8)
	No (B)	Mean (SD)	60.8 (28.7)	52.7 (27.5)	53.5 (24.5)	33 (9.1)	33.9 (9.6)	34.4 (7.7)	43.5 (13.1)	47 (13.7)	47.3 (10.6)
		95% CI	(49.6 - 72.1)	(41.9 - 63.4)	(43.3 - 63.7)	(29.4 - 36.5)	(30.2 - 37.7)	(31.2 - 37.7)	(38.4 - 48.7)	(41.6 - 52.4)	(42.9 - 51.7)
3	Yes (A)	Mean (SD)	61.9 (25.2)	56.2 (21)	65.8 (19)	31.9 (9)	33.1 (7.9)	31.8 (9.3)	48.9 (11.2)	49 (11)	44 (12.7)
		95% CI	(51.8 - 72)	(47.8 - 64.6)	(58 - 73.5)	(28.3 - 35.5)	(30 - 36.3)	(27.9 - 35.6)	(44.4 - 53.4)	(44.6 - 53.3)	(38.8 - 49.2)
	No (B)	Mean (SD)	46.8 (28.3)	53.7 (24.1)	51.5 (13.8)	34.7 (7.7)	36.1 (10.3)	37.7 (9.2)	46.8 (9.9)	49.7 (10)	49.7 (9.1)
		95% CI	(35.7 - 57.8)	(44.2 - 63.1)	(45.9 - 57.1)	(31.7 - 37.7)	(32.1 - 40.2)	(33.9 - 41.5)	(42.9 - 50.6)	(45.7 - 53.6)	(45.9 - 53.4)
4	Yes (A)	Mean (SD)	53 (25.8)	59.8 (26.7)	63.5 (18)	33.6 (7.7)	32.7 (8.1)	32.8 (7.3)	45.4 (12.1)	48.6 (17)	45.5 (11.1)
		95% CI	(42.4 - 63.5)	(48.9 - 70.7)	(55.9 - 71)	(30.4 - 36.7)	(29.4 - 36)	(29.8 - 35.9)	(40.5 - 50.4)	(41.7 - 55.6)	(40.9 - 50.2)
	No (B)	Mean (SD)	61.7 (28.7)	62 (21.5)	61.2 (19.2)	33.7 (7.5)	34.6 (8.6)	34.5 (8.1)	43.3 (13.3)	43.6 (13.9)	41.4 (13.1)
95% CI	(50.2 - 73.1)	(53.4 - 70.6)	(53 - 69.4)	(30.7 - 36.7)	(31.2 - 38)	(31.1 - 38)	(38 - 48.7)	(38 - 49.1)	(35.7 - 47)

SD: Standard Deviation / CI: Confidence Interval / VAS: Visual Analog Scale; SF-36 PCS: Medical Outcomes Study - 36 Itens Short Form Health Survey - Physical Component Summary; SF-36 MCS: Medical Outcomes Study - 36 Itens Short Form Health Survey - Mental Component Summary.

The relative proportions of subgroups were maintained when analysing the Class and Control groups with 76.4% and 76.6% women, 62.8% and 68.1% of white race and 68.9% and 72.3% bilaterality, respectively. Both groups had similar percentages of K&L grades II, III and IV (for the Class group: right knee: 33.1%, 37.2% and 20.9%; left knee: 33.3%, 38.8% and 19%, respectively; for the Control group: right knee: 29.5%, 31.8% and 36.4%; left knee: 31.9%, 42.6% and 17%, respectively, p=0.22).

Table 5 shows the results of the BMI, PBF, pain and functional questionnaires and functional tests from the Class and Control groups. BMI remained the same timewise in both groups (p=0.52 and p=0.46, respectively). PBF remained similar in both groups but changed timewise in both groups (p=0.46 and p=0.001, respectively). The Lequesne questionnaire results were initially similar between groups and later improved in the Class group and worsened in the Control group (p=0.02 at two years and p<0.001, timewise). TUG and FTSST also showed differences timewise (p=0.01 and p<0.001, respectively).

**Table 5:** Descriptions of anthropometric measures, pain and functional scales and functional tests (TUG and FTSST) according to subgroups and moments.

		Group Class			Group Control			Significance	
		Baseline	1 year	2 years	Baseline	1 year	2 years	Between Groups	Time
								p	p
BMI	Mean (SD)	31.29 (5.41)	31.14 (5.37)	31.44 (5.33)	31.30 (5.19)	31.15 (5.79)	31.44 (6.18)	0.52	0.46
	CI	30.14 - 32.37	30.11 - 32.30	30.26 - 32.35	29.48 - 33.96	29.00 - 34.04	28.51 - 33.64		
BFP	Mean (SD)	36.26 (8.46)	35.75 (8.56)	38.34 (8.39)	36.26 (8.45)	35.96 (8.11)	37.73 (8.49)	0.46	0.001*
	CI	34.32 - 37.87	33.94 - 37.56	36.37 - 40.06	33.92 - 41.05	33.80 - 40.68	33.40 - 42.64		
Womac	Mean (SD)	46.07 - (18.31)	42.85 (17.22)	42.01 (18.32)	43.51 (18.14)	46.15 (19.42)	43.91 (15.02)	0.74	0.47
	CI	40.42 - 48.80	38.85 - 46.27	35.57 - 43.26	36.61 - 48.49	34.05 - 50.85	34.62 - 48.18		
Womac Pain	Mean (SD)	9.11 (4.06)	8.23 (3.67)	8.47 (3.91)	8.00 (4.12)	9.00 (4.17)	9.00 (3.33)	0.40	0.68
	CI	8.09 - 9.88	7.44 - 9.04	7.15 - 8.77	7.32 - 10.68	6.81 - 9.99	6.56 - 9.74		
VAS	Mean (SD)	58.55 (26.58)	53.39 (23.47)	55.31 (21.42)	57.4 (27.36)	60.89 (23.96)	62.35 (18.45)	0.14	0.88
	CI	51.44 - 62.75	47.01 - 56.98	46.27 - 55.20	45.84 - 69.76	41.05 - 65.45	50.32 - 68.28		
Lequesne	Mean (SD)	11.81 (4.09)	11.76 (4.02)	11.51 (4.39)	11.97 (3.71)	12.26 (4.06)	12.16 (4.13)	0.02	<0.001*
	CI	10.61 - 12.48	10.46 - 12.18	9.81 - 11.72	9.61 - 13.34	9.98 - 13.37	9.45 - 13.25		
Time-Up-and-Go	Mean (SD)	12.2 (4.42)	12.08 (4.37)	11.79 (4.87)	13.71 (6.00)	12.6 (4.73)	12.6 (5.20)	0.31	0.01*
	CI	11.11 - 13.23	10.95 - 12.83	10.29 - 12.09	11.63 - 16.40	10.92 - 15.66	9.89 - 15.15		
Five-Times-Sit-to-Stand	Mean (SD)	23.23 (8.26)	18.17 (5.96)	19.43 (6.65)	22.79 (11.08)	19.66 (10.26)	23.24 (10.49)	0.13	<0.001*
CI		21.26 - 25.13	16.71 - 19.13	18.13 - 20.84	18.10 - 29.69	15.42 - 26.53	18.08 - 29.76		

SD: Standard Deviation; CI: Confidence Interval; BMI: Body Mass Index; BFP: Body Fat Percentage; VAS: Visual Analog Scale.

The intensity of physical activity was similar between groups prior to the program. At the end of the study, the Class group had incorporated more physical activity into their weekly program than the Control group had (p=0.45, Table 6).

**Table 6:** Description of the intensity of physical activity weekly practiced according to subgroups and moments of evaluation and results of the comparative tests.

	Baseline				2 years		
	Group Class		Group Control		Group Class		Group Control	
Intensity of physical activity	n	%	n	%	n	%	n	%
Does not perform	118	84.9%	40	90.9%	38	27.3%	22	50.0%
Light activity	7	5.0%	4	9.1%	60	43.2%	14	31.8%
Moderate activity	12	8.6%	0	0.0%	33	23.7%	7	15.9%
Vigorous activity	2	1.4%	0	0.0%	8	5.8%	1	2.3%
	p = 0.139				p = 0.045		

Chi-squared test.

## DISCUSSION

Individuals over 60 years of age represented 8.6% of the Brazilian population in 2000 (which was 169,799,170), and the projection for 2030 was 13.44% of all Brazilians (223,126,917).^16^ ^,^ ^17^ The population of the United States in 2010 was 308,745,538, with 13% individuals over 65 years of age. In that same year, in the United States, total knee replacement was the most frequently performed inpatient procedure on adults aged 45 and over.^18^ Between 2000 and 2010, an estimated 5.2 million total knee replacements were performed, and adults over 45 years old comprised 98.1% of those surgeries.^19^

Regardless of the low education and socioeconomic status of the Brazilian population, increasing longevity and obesity (74.1% of people over 65 years of Brazil were overweight or obese in 2008-2009) perpetuate the increasing prevalence of OA and a low quality of life.^3^ ^-^ ^6^ ^,^ ^20^

The aim of this educational program on OA was to teach patients about the nature, causes and treatment of osteoarthritis and, above all, to improve patients' knowledge and health behaviour. The one-year results of the program led to the suspension of bimonthly telephone calls. Telephone calls were time consuming and were not effective in modifying the adherence to the diet program, exercise and social engagement.^7^

Our subgroups and groups were homogeneous as to the degree of KOA (70% grades 2 and 3, and 20% grade 4 K&L), age (average age 64.4 years), gender (approximately 3 women to 1 man), race (60% Caucasians), PBF (approximately 36%), affected side (80% affected the right side) or bilateral (70% with bilateral involvement), and questionnaire results (subjective) (p>0.05, Tables 1 and 2). Table 2 shows that subgroups 2 and 3 differed in BMI (p = 0.049) but not in PBF (p = 0.421). When we consider the Class and Control groups, the groups were similar in all parameters (p> 0.05). Tables 3 and 4 show the results of the pain, function and quality of life questionnaires of subgroups 1 to 4 (still registering those who received bimonthly phone calls in the first year of the study). The differences in the results were not significant, as was expected.^7^ ^,^ ^8^

When comparing the Class and Control groups (Table 5), both groups failed to lose the minimum 6.1 kg for symptomatic improvement (10), but although the Class group improved or maintained functional outcomes in the Lequesne questionnaire, the group that received the educational material only (Control) progressively worsened their Lequesne results (p=0.02 between groups, p<0.001 over time). Objective tests of TUG and TSL that represent the strength of lower limbs and balance^13^ ^,^ ^14^ improved over time, especially in the Class group.

Both the Class and Control groups were similar with respect to physical activity practiced at baseline (Table 6), but the number of participants who incorporated physical activity and at greater intensity was significantly higher in the group that joined classes (p=0.045), thus reinforcing the increased strength and balance observed by the objective TUG and FTSST tests and by the subjective Lequesne questionnaire results.

Our educational program failed to significantly reduce the BMI of the participants (Tables 3-5). Roughly one-third lost weight (at least 1 point in BMI), one-third remained at a similar weight and the last third gained weight. Because obesity and OA yield substantial losses in quality-adjusted life-years,^20^ this deficiency in the program needs to be rectified. The project may have raised the awareness of the need for physical activity and diet, but only 12% actually lost more than 2 points in BMI (6.1 kg for a person 1.75 m tall). The increase in physical activity and improved function were the main effects of the educational program.

## CONCLUSION

The educational program with classes improved the performance of physical activity and both subjective and objective function of patients with KOA.
